# Diagnosis and therapeutic monitoring of inborn errors of metabolism in 100,077 newborns from Jining city in China

**DOI:** 10.1186/s12887-018-1090-2

**Published:** 2018-03-13

**Authors:** Chi-Ju Yang, Na Wei, Ming Li, Kun Xie, Jian-Qiu Li, Cheng-Gang Huang, Yong-Sheng Xiao, Wen-Hua Liu, Xi-Gui Chen

**Affiliations:** 1 Center of Neonatal Disease Screening, Maternal and Child Health Care Hospital, Number 12, Gongxiao Road, Jining, Shandong Province People’s Republic of China; 2 Clinical Laboratory of Linyi People’s Hospital, Linyi, Shandong Province People’s Republic of China; 3 Zhejiang Biosan Biochemical Technologies Co., Ltd., Hangzhou, Zhejiang Province People’s Republic of China

**Keywords:** Tandem mass spectrometry, Inborn errors of metabolism (IEM), Aminoacidemia, Organic acidemia, Fatty acid oxidation disorders

## Abstract

**Background:**

Mandatory newborn screening for metabolic disorders has not been implemented in most parts of China. Newborn mortality and morbidity could be markedly reduced by early diagnosis and treatment of inborn errors of metabolism (IEM). Methods of screening for IEM by tandem mass spectrometry (MS/MS) have been developed, and their advantages include rapid testing, high sensitivity, high specificity, high throughput, and low sample volume (a single dried blood spot).

**Methods:**

Dried blood spots of 100,077 newborns obtained from Jining city in 2014-2015 were screened by MS/MS. The screening results were further confirmed by clinical symptoms and biochemical analysis in combination with the detection of neonatal deficiency in organic acid, amino acid, or fatty acid metabolism and DNA analysis.

**Results:**

The percentages of males and females among the 100,077 infants were 54.1% and 45.9%, respectively. Cut-off values were established by utilizing the percentile method. The screening results showed that 98,764 newborns were healthy, and 56 out of the 1313 newborns with suspected IEM were ultimately diagnosed with IEM. Among these 56 newborns, 19 (1:5267) had amino acid metabolism disorders, 26 (1:3849) had organic acid metabolism disorders, and 11 (1:9098) had fatty acid oxidation disorders. In addition, 54 patients with IEM were found to carry mutations, and the other 2 patients had argininemia.

**Conclusions:**

Fifty-six cases of metabolic disorders in Jining were confirmed via newborn screening (NBS) by MS/MS. Early diagnosis and treatment are crucial for the survival and well-being of affected children. A nationwide NBS program using MS/MS is recommended, especially in poor areas of China.

## Background

Newborn screening (NBS) is a public health program for the screening of infants shortly after birth for a list of conditions that are treatable but not clinically evident in the newborn period that could cause severe illness or death without early detection and treatment [1]. The NBS program aims to diagnose infants born with certain genetic, metabolic and functional disorders [2]. By early diagnosis and treatment, severe neurologic damage can be avoided in advance. NBS is designed to provide early diagnosis and treatment before significant and irreversible damage occurs [3, 4].

With the increasing number of disorders for which screening methods are available, there is a greater need for clinical diagnosis and differential diagnosis of genetic metabolic disorders. Traditional screening methods have not met this need because their speeds of testing were slow and their false positive rates were relatively high, greatly limiting their suitability for large-scale neonatal screening [5]. In the 1980s, tandem mass spectrometry (MS/MS) was introduced into newborn screening. The use of MS/MS in newborn screening makes it possible to screen for a wide range of previously unscreened inborn errors of metabolism (IEM) using a single test [6]. The disorder profile of IEM includes aminoacidemias, fatty acid oxidation (FAO) disorders and organic acidemias. Early screening and diagnosis may help to decrease mortality and morbidity rates in children with IEM. Tandem mass spectrometry (MS/MS) techniques are now routinely used to screen more than 30 types of IEM, including aminoacidopathies, organic acid disorders and fatty acid oxidation disorders, given their advantages such as rapid testing, high sensitivity, high specificity, high throughput and a low sample volume (a single blood spot). By simply analyzing a dry blood spot (DBS), MS/MS was able to provide significant technical support to the diagnosis of IEM by detecting the characteristics of amino acids and acylcarnitines, thereby facilitating the screening, diagnosis and treatment of IEM [7, 8].

China is the world’s most populous country. There are approximately 20 million newborns in China every year, among whom 400 to 500 thousand have inherited metabolic disorders, and the incidence of these disorders is very high [9]. From 2000 to 2009, 1, 495,132 neonates were screened in Taiwan, among whom 170 patients were diagnosed with an inherited metabolic disorder. The total incidence rate was 1:6219, including 107 cases of amino acid metabolism disorder, 51 cases of organic acid metabolism disorder and 12 cases of fatty acid metabolism disorder [10]. In the mainland, Xinhua Hospital in Shanghai introduced MS/MS technology into diagnosis of IEM in 2003. Since then, a total of 269,341 newborns have been screened, in whom 14 types of inherited metabolism disorders were detected, totaling 74 cases with an incidence rate of 1:3640. Subsequently, the neonatal screening center of Guangzhou City began to use tandem mass spectrometry to provide screening services for 30 types of inherited metabolic disorders in 2007. The neonatal screening center of Zhejiang Province provided screening for 26 kinds of genetic metabolic diseases with tandem mass spectrometry in 2008 and expanded this program to newborn screening in 2009. From 2009 to September 2010, approximately 129,415 newborns were screened. In total, 13 types of disorders of amino acids, organic acids and fatty acid metabolism were detected, for a total of 23 cases with an incidence rate of 1:5626. More than 50 screening centers for neonatal diseases have carried out the program in China [11].

However, because of economic and technological limitations, the expansion of the NBS program in China has been slow. Jining, one of the most populated cities in Shandong Province in China, carried out a program to screen for inherited metabolic disorders in newborns with MS/MS, aiming at improving the health of the population and the potential of clinical medicine. In 2014, the Jining municipal government added newborn screening for inherited metabolic disorders by tandem mass spectrometry to the city’s birth defects tertiary prevention engineering and provided funding for the screening costs to promote the application of this technology.

With MS/MS newborn screening, a variety of common genetic metabolic disorders can be detected simply. The occurrence of disability or death and other serious consequences of IEM in children can be avoided by early detection and timely treatment, thus improving the health of the newborns and reducing the incidence of birth defects in local areas, as well as decreasing the economic burden on patients, families and society.

In this study, 100,077 newborns attended the screening, and we report the estimated incidences of inborn errors of amino acid, organic acid and fatty acid metabolism in suspected children referred for screening in Jining city.

## Methods

DBS samples were collected from 100,077 (54,141 male and 45,936 female) infants in Jining city between 2014 and 2015. Each DBS was collected by sticking the inside or outside heel edge and then drawing a few drops of blood on filter paper (United States S&S903). The blood was allowed to thoroughly saturate the paper, and the sample was air dried for several hours. The filter paper was then sent to the neonatal center by cold chain transportation within 5 working days. The Ethics Committee of Jining Maternal and Child Health Care Hospital & Ethics Committee of Shandong province approved the study protocol (authorization number: JNCH-0128).

### Reagents

The analysis was performed using a commercially available LC-MS/MS kit permitted for sale in the Chinese market (NeoBase™ Non-derivatized MS/MS Kit, Perkin Elmer, MA, USA).

### Pretreatment of DBS

A 3.2-mm-diameter disc of saturated paper was separated from the filter paper and dropped into a 96-well U-bottomed plate. For extraction, 100 μL of extraction solution (containing a mixture of the respective stable-isotope-labeled internal standard) was added to each well. The plate was sealed by viscous film and incubated for 45 min at 45 °C on a horizontal orbital microplate shaker set at 650 rpm. The extracted solutions were transferred to a V-bottomed plate and covered with aluminum foil. Then, the samples were incubated at room temperature for 2 h. Finally, the plate was placed in the autosampler for testing.

Eleven amino acids, specifically, alanine (ALA), citrulline (CIT), arginine (ARG), glycine (Gly), leucine/isoleucine (Leu/lle/Pro-OH), methionine (MET), ornithine (ORN), phenylalanine (PHE), proline (PRO), tyrosine (Tyr), and valine (VAL); 31 acylcarnitines, namely, C0, C2, C3, C3DC/C4OH, C4, C4DC/C5OH, C5, C5:1, C5DC/C6OH, C6, C6DC, C8, C8:1, C10, C10:1, C10:2, C12, C12:1, C14, C14:1, C14:2, C14OH, C16, C16:1, C16OH, C16:1OH, C18, C18:1, C18:2, C18OH, and C18:1OH; and one ketone, succinylacetone (SA), were analyzed.

### Apparatus settings

Acylcarnitines and amino acids extracted from each DBS were analyzed on a triple quadrupole tandem mass spectrometer equipped with an ACQUITY TQ Detector (Waters, MA, USA) and coupled to a Waters 1525 binary HPLC pump. Mass spectrometry was performed in multiple reaction monitoring (MRM) mode. The abundance of each compound was quantified by calculating the signal intensity ratio of the compound to its internal standard.

### Confirmation of diseases

The diagnostic procedure was as follows:

1. The MS/MS test was repeated, and the results were compared to the data from the first test. 2. The levels of urinary organic acids were determined by GC-MS, or high-precision DNA mass spectrometry analysis was performed for the 39 genetic mutations related to the clinical disorders of neonatal organic acid, amino acid, and fatty acid metabolism.

## Results

### Basic characteristics

Among the 100,077 newborns from whom screening samples were collected, the 54,141 males accounted for 54.1% of the cohort, and the 45,936 females accounted for 45.9%. The age of blood collection from the newborns ranged from 3 to 37 days, with an average age of (5.2 ± 3.8) days.

### Analysis of the screening results

According to the neonatal screening results, 98,764 infants were healthy, and 1313 were suspected to have IEM (shown in Table 1), for a positive rate of 1.31%. Among these 1313 suspected cases, 1152 were recalled for retesting (87.74%), with the final results being negative, and the remaining 161 (12.26%) cases were recalled for further diagnostic assessment. Ultimately, 56 cases of IEM were confirmed.

**Table 1:** MS/MS screening profiles

MS/MS analytes	Cut-off value (μmol/L)	Possible disorder(s)	Number of cases	Ratio
Amino acids				
↑PHE	> 100	Phenylketonuria	29	2.21%
↑PHE/TYR	> 1.5			
↑MET	> 55	Hypermethioninemia	53	4.04%
↑MET/PHE	> 0.95	Cystathionine-γ-lyase deficiency	36	2.74%
↑LEU	> 300	Maple syrup urine disease	14	1.07%
↑LEU/PHE	> 5.46			
↑VAL	> 300			
↑TYR	> 290	Tyrosinemia	101	7.69%
↑TYR/PHE	> 7			
↑SA	> 1	Tyrosinemia type I	22	1.68%
↑CIT	> 40	Citrullinemia	22	1.68%
↑CIT/PHE	> 0.7			
↑ARG	> 60	Argininemia	37	2.82%
↓CIT	< 7	Carbamoyl-phosphate synthase deficiency	139	10.59%
↑ORN	> 350	Hyperornithinemia	27	2.06%
↑PRO	> 420	Hyperprolinemia	30	2.28%
Organic Acids				
↑C3	> 4	Methylmalonic acidemia	121	9.22%
↑C3/C2	> 0.17	Propionic acidemia	34	2.59%
↑C3DC + C4OH	> 0.35	Malonic acidemia	15	1.14%
↑C4	> 0.45	Glutaric acidemia type II	2	0.15%
↑C5	> 0.43	Isovaleric acidemia	138	10.51%
↑C5/C2	> 0.04			
↑C5DC + C6OH	> 0.2	Glutaric acidemia type I	1	0.08%
↑C4DC + C5OH	> 0.4	3-methylcrotonyl-CoA carboxylase deficiency	14	1.07%
		Multiple carboxylase deficiency	90	6.85%
↑(C4DC + C5OH)/C8	> 11	3-OH-3-methylglutaryl-CoA lyase deficiency	12	0.91%
↑C5:1	> 0.02	Beta-ketothiolase deficiency	9	0.69%
↑C8	> 0.15			
Fatty acid oxidation disorders				
↓C0	< 10	Carnitine uptake defect	216	16.45%
↑C0	> 50	Carnitine palmitoyltransferase I deficiency	10	0.76%
↑C0/(C16 + C18)	> 45			
↓C16	< 0.45			
↑C4	> 0.45	Short-chain acyl-CoA dehydrogenase deficiency	24	1.83%
↑C4/C2	> 0.03	Ethylmalonic encephalopathy	65	4.95%
↑C8	> 0.15	Medium-chain acyl-CoA dehydrogenase deficiency	22	1.68%
↑C8/C10	> 1.5			
↑C6	> 0.1			
↑C10:1	> 0.15			
↑C14:1	> 0.25	Very long-chain acyl-CoA dehydrogenase deficiency	9	0.69%
↑C14:1/C16	> 0.09			
↑C14	> 0.4			
↑C16	> 6.5	Carnitine palmitoyltransferaseII deficiency	3	0.23%
↑C18	> 1.9	Carnitine-acylcarnitine translocase deficiency	5	0.38%
↑C18:1	> 3.5			
↑C16OH	> 0.04	Long-chain hydroxyacyl-CoA dehydrogenase deficiency	4	0.30%
↑C18OH	> 0.03	Trifunctional protein deficiency	9	0.69%
↑C18:1OH	> 0.05			

### Analysis of diagnosed diseases

From the results of further confirmatory tests, 56 out of the 1313 suspected cases were finally diagnosed with IEM. Among these 56 infants, 19 (1:5267) had amino acid disorders, 26 (1:3849) had organic acid disorders, and 11 (1:9098) had fatty acid oxidation disorders. Moreover, 54 of these individuals carried mutations, while the other 2 patients had argininemia.

### Aminoacidemias

Three types of aminoacidemias were detected in this study (16 cases with PKU, 2 cases with argininemia and 1 case with cystathionine γ-lyase deficiency), and the total prevalence rate was 1:5267. The data also indicated that PKU was the most common amino acid disorder in Jining city, as it is in other provinces in China. Other aminoacidemias were rare, such as the remaining two types, argininemia (2 patients, 10.5%) and cystathionine γ-lyase deficiency (1 patient, 5.3%) (Table 2). In the patients with PKU, the Phe level in blood was highly elevated, accompanied by an increase in the Phe/Tyr ratio. According to the confirmatory test, the gene mutation of phenylalanine hydroxylase (PAH) was the main cause of PKU. For the 2 cases of argininemia, no mutated genes were found. For the 1 case of cystathionine γ-lyase deficiency, a gene mutation of MAT1A led to this disease.

**Table 2:** Abnormal MS/MS results of aminoacidemias

Aminoacidemias (*n* = 19)	n (%)	Age at Diagnosis (day)	Abnormal parameter	Concentration mean (range)(μmol/l)	Reference range (μmol/l)	Mutated gene
Phenylketonuria (PKU)	16(84.2)	10-40	Phe	987.39 (139.92-2607.82)	21-100	PAH, PTS
			Phe/Tyr	15.66 (1.75-37.76)	0.12~ 1.1	
Argininemia (ARG)	2(10.5)	41-48	Arg	92.24 (73.97-110.50)	1.5-65	–
Cystathionine-γ-lyase deficiency	1(5.3)	35	MET	87.97	11-45	MAT1A
			MET/PHE	2.81	0.2-0.8	

### FAO disorders

There were 11 infants diagnosed with FAO disorders, for a total prevalence rate of 1:9098 (eight cases of primary carnitine deficiency [PCD], two cases of medium-chain acyl-CoA dehydrogenase [MCAD] deficiency, and one case of short-chain acyl-CoA dehydrogenase [SCAD] deficiency) (Table 3). PCD was caused by mutation of the *SLC22A5* gene, with a low blood C0 level as a hallmark. Patients with MCAD deficiency can be identified based on high blood C8 levels, which is due to mutation of the *ACADM* gene. SCAD deficiency is caused by mutation of the *ACADS* gene. This disease can be confirmed by an elevated blood C4 level and by high ethylmalonic acid levels in urine.

**Table 3:** Abnormal MS/MS results of fatty acid oxidation disorders

Fatty acid oxidation disorders (*n* = 11)	n (%)	Age at Diagnosis (day)	Abnormal parameter	Concentration mean (range)(μmol/l)	Reference range (μmol/l)	Mutated gene
Primary carnitine deficiency(PCD)	8(72.7)	25-76	C0	4.32(2.40-6.43)	10-55	SLC22A5
Medium-chain acyl-CoA dehydrogenase (MCAD)	2(18.2)	45-49	C8	2.07(0.63-3.51)	0.01-0.15	ACADM
			C10	0.18(0.15-0.21)	0.02-0.22	
Short-chain acyl-CoA dehydrogenase (SCAD)	1(9.1)	102	C4	1.94	0.07-0.45	ACADS

### Organic acidemias

The most common organic acidemias in this group of patients was methylmalonic acidemia (MMA, 16, 59.3%), followed by 3-methylcrotonyl CoA carboxylase (3-MCC) deficiency (5, 18.5%), ethylmalonic encephalopathy (EE, 4, 14.8%) and β-ketothiolase deficiency (BKD, 1, 3.7%) (Table 4). Two types of gene mutations were confirmed from the blood samples of patients with MMA: MMACHC and MUT. Almost all patients with MMA (14/16, 88%) were in the group with MMACHC gene mutations. The confirmatory tests showed that methylmalonic acidemia and homocysteinemia resulted from a defect in the steps leading to adenosylcobalamin (AdoCbl) synthesis. In the remainder of the patients with MMA, the disorder was confirmed to result from a defect in the apoenzyme L-methylmalonyl-CoA mutase (MCM) due to a mutation of the MUT gene. Aside from MMA, other organic acidemias were rare. The number of patients with EE was 4. The diagnostic marker of EE was similar to that of SCAD deficiency, which can be distinguished by analyzing the organic acid content in urine. In patients with EE, the ethylmalonic acid level was elevated. Infants with 3-MCC, the 3-hydroxyisovaleric acid and 3-methylcrotonyl glycine levels in urine were specifically elevated. Regarding BKD, which was caused by gene mutation of ACAT1, 2-methyl-3-hydroxyl butyric acid and methylcrotonyl glycine levels in urine showed specific increases.

**Table 4:** Abnormal MS/MS results of organic acidemias

Organic acidemias (*n* = 26)	n (%)	Age at Diagnosis (day)	Abnormal parameter	Concentration mean (range)(μmol/l)	Reference range (μmol/l)	Mutated gene
Methylmalonic academia (MMA)	16 (61.6)	13-121	C3	7.50 (4.27-18.94)	0.28-4.3	MMACHC, MUT
			C3/C2	0.90 (0.24-1.85)	0.02-0.17	
Ethylmalonic encephalopathy (EE)	4 (15.4)	30-47	C4	1.47 (1.22-1.64)	0.07-0.45	ACADS
			C4/C3	1.59 (1.47-1.77)	0.04-0.4	
3-methylcrotonyl CoA carboxylase deficiency (3-MCC)	5 (19.2)	21-57	C4DC + C5OH	6.36 (0.94-14.94)	0.06-0.4	MCCC1
β-ketothiolase deficiency (BKD)	1 (3.8)	37	C5:1	0.47	0-0.02	ACAT1

## Discussion

Early detection and treatment for IEM can effectively prevent or reduce serious developmental and neurological damage. MS/MS is a useful tool for diagnosing different types of IEM, such as organic acidemias or aminoacidopathies [12–14]. With its feature of high throughput, MS/MS is suitable both for diagnosis of patients in the clinic and for newborn screening [7, 15]. Although the MS/MS method has been used for diagnosis for many years in China, its use has mainly been concentrated in high-end laboratories of developed areas [16]. NBS by MS/MS is not currently a national project, and corresponding supporting policy is lacking. For some poor areas that cannot afford the cost of diagnostic screening, there is also a lack of data regarding NBS. Therefore, it is difficult to establish an effective cut-off value of the screening index for guidance of the screening of IEM. In this study, 100,077 infants in Jining city underwent screening for IEM, and laboratory screening cut-off values were established based on the results. The percentage of infants with IEM was approximately 0.56‰ (56 cases were confirmed), which was lower than the positive detection rate in the other regions of Asia [17, 18], probably because of different screening criteria, screening areas or sample collection methods [18, 19].

Amino acid disorders accounted for 33.9% of all cases of IEM, and PKU was the most common type. Except for one HPA case, which was caused by mutation of the *PTS* gene, the HPA cases were related to mutation of the *PAH* gene. The rate of PKU was 50% below that of the entire country, which may be because PKU patients in some areas were not detected at an early age. According to our data, the median age of PKU patients was 16 days (range: 10-40 days). Once PKU diagnosis was confirmed, therapy was initiated, a strict diet with measures such as drinking a special formula was implemented, and regular checks were conducted to confirm that the phenylalanine concentration in blood remained in the range of 120~ 600 μmol/L. With effective treatment, the physical and mental development of the PKU patients in our study were equivalent to those of healthy children of the same age.

By analyzing the concentration of Arg in blood and urine, two cases of argininemia were confirmed, although the associated gene mutation was still unknown. The MS/MS screening results showed that patients with cystathionine-γ-lyase deficiency had higher Met levels and Met/Phe ratios. By detecting the amino acids in plasma, we observed that the concentrations of methionine and cystathionine were high, while the cysteine concentration was lower than in normal samples. The c.791G > A mutation in the *MAT1A* gene was found to be responsible for this disorder.

According to other studies, FAO disorders were not common in the Asian population [20, 21], and our data showed that FAO disorders accounted for 17.9% of all IEM cases. PCD was the most common type of FAO disorder in this study, and patients were identified between 1 month and 3.5 months of age. With oral supplementation of carnitine and avoidance of hunger, patients with PCD were not different from normal babies. Only two MCAD cases were found in this study and were caused by the c.449-452delCTGA mutation in the *ACADM* gene. The remaining case was SCAD deficiency with the c.1031A > G mutation in the *ACADS* gene. Other types of FAO disorders are also rare in the Chinese population. However, blood sampling was not performed under strictly fasting conditions for most infants, and patients with some types of FAO disorders may have been missed, thus underestimating the incidence of FAO disorders.

Organic acidemias accounted for 48.2% of IEM cases in this study, and MMA, 3-MMC and EE were the three most common types. The patients with MMA accounted for 61.6% (16 patients) of the patients with organic acidemias, consistent with the findings in other countries [22–24] that also indicated MMA as the most common organic acidemia. In the patients with MMA, there were two types of therapy, depending on whether vitamin B_12_ (VitB_12_) treatment was effective. Fortunately, VitB_12_ was useful for most of the patients, except for one with a mutation in the *MUT* gene. For the one patient, the successful method was diet therapy with a special formula removing several natural amino acids, such as Leu and Val. With the therapy, most were normal, except that one had motor disorders because of the parents stopping VitB_12_ treatment of their own accord. It was a pity for the family, as they knew little about the damage of inherited metabolic disorders. This experience serves as an alarm for us to consider not only the technology but also the need for people to understand the damage of IEM. Additionally, the program could provide genetic counseling for families with babies suffering from IEM.

Other organic acidemias are relatively rare but also lead to physical or nerve damage. The patients with EE had persistent lactic acidosis. The biochemical characteristics are typical of ethylmalonic aciduria, with a high concentration of methylsuccinic acid in urine and a high C4 concentration and C4/C3 ratio in blood. The patients with 3-MMC presented hypoglycemia with low ketone levels, sleepiness and vomiting. The BKD patient suffered from intermittent ketoacidosis accompanied by sleepiness and even coma. With early diagnosis and treatment, infants with IEM grew in a healthy state. Our study indicated that if patients with organic acidemias were not diagnosed and treated at an early stage of the disorder, irreversible sequelae of the nervous system usually occurred. In addition, except for 2 cases with argininemia, the remaining 54 patients had gene mutations that were detected. Among the 54 patients with gene mutations, 6 patients carried homozygous mutations (5 patients with primary carnitine deficiency, 1 with methylmalonic acidemia), and the remaining 48 carried heterozygous mutations. Except for one mutation in the *PTS* gene, the gene mutations of PKU patients were centered on the *PAH* gene. In total, 13 types of mutations in the *PAH* gene and 2 in the *PTS* gene were detected. Almost every patient with PKU carried unique gene mutations, which indicated that the gene mutations causing PKU were diverse, as in patients with MMA. In total, 11 types of mutations in the *MMACHC* gene and 2 in the *MUT* gene were detected, with c.609G > A being the most frequent mutation. PCD was the third most common disorder related to IEM. By DNA analysis, the gene mutation of all patients was c.1400C > G in the *SLC22A5* gene.

## Conclusions

Extensive newborn screening by the MS/MS method is important for early diagnosis and effective treatment of IEM. Because people in different areas may have different blood indices for many factors, establishing a specific cut-off value for quickly and accurately diagnosing IEM is necessary. In this study, IEM could be accurately diagnosed by the combination of the MS/MS method and DNA analysis. Based on the established methods of newborn screening in Jining city, other areas of China, especially some poor areas, can obtain effective clinical guidance for the diagnosis of IEM. This guidance is helpful for saving the lives of newborns and reducing the governmental economic burden.

## Abbreviations

AdoCbl: Adenosylcobalamin
ALA: Alanine
ARG: Arginine
BKD: β-ketothiolase deficiency
CIT: Citrulline
DBS: Dry blood spot
EE: Ethylmalonic encephalopathy
FAO: Fatty acid oxidation
GLY: Glycine
HPA: Hyperphenylalaninemia
HPLC: High-performance liquid chromatography
IEM: Inborn errors of metabolism
MCAD: Medium-chain acyl-CoA dehydrogenase
MCM: L-methylmalonyl-CoA mutase
MET: Methionine
MMA: Methylmalonic acidemia
MRM: Multiple reaction monitoring
MS/MS: Tandem mass spectrometry
NBS: Newborn screening
ORN: Ornithine
PCD: Primary carnitine deficiency
PHE: Phenylalanine
PKU: Phenylketonuria
PRO: Proline
SA: Succinylacetone
SCAD: Short-chain acyl-CoA dehydrogenase
TYR: Tyrosine
VAL: Valine
VitB12: Vitamin B12
